# Supplementary material for: Synthesis and Molecular Docking of Some Novel 3-Thiazolyl-Coumarins as Inhibitors of VEGFR-2 Kinase
Source: Molecules. 2023 Jan 10;28(2):689. doi: 10.3390/molecules28020689 (PMC9861390; doi:10.3390/molecules28020689)
Supplement: Supplementary file 1 [file molecules-28-00689-s001.zip › molecules-2101283-supplementary.pdf]

Supplementary File S1

Synthesis and Molecular Docking of Some Novel 3-Thiazolyl-Coumarins as Inhibitors of VEGFR-2 Kinase

Tariq Z. Abolibda [1], Maher Fathalla[1,2], Basant Farag[2] , Magdi E. A. Zaki[3] and Sobhi M. Gomha[1,4,*],

1Department of Chemistry, Faculty of Science, Islamic University of Madinah, Madinah 42351, Saudi Arabia

2Department of Chemistry, Faculty of Science, Zagazig University, Zagazig 44519, Egypt

3Department of Chemistry, Faculty of Science, Imam Mohammad Ibn Saud Islamic University (IMSIU), Riyadh 11623, Saudi Arabia

4Department of Chemistry, Faculty of Science, Cairo University, Cairo 12613, Egypt

*Correspondence: smgomha@iu.edu.sa or s.m.gomha@cu.edu.eg or s.m.gomha@gmail.com

Experimental

Melting points were detected using an Electrothermal IA 9000 series digital melting point instrument. Using infrared spectrophotometers such as the Pye Unicam SP 3300 and Shimadzu FTIR 8101 PC, IR spectra of potassium bromide discs were captured. ^{1}H-NMR and ^{13}C-NMR spectra were recorded in DMSO solutions using a BRUKER 400 FT-NMR spectrometer, and chemical shifts were expressed in ppm using TMS as an internal reference. The Shimadzu GCeMS-QP1000 EX mass spectrometer was used to record mass spectra at 70 eV. Elements were analyzed using an Elementar Vario LIII CHNS analyzer manufactured in Germany. The antitumor activity was evaluated by the Regional Center for Mycology and Biotechnology of Al-Azhar University, in Cairo, Egypt.

3-((1-(2-(4-Methyl-5-(phenyldiazenyl)thiazol-2-yl)hydrazineylidene)ethyl)-6-(phenyldiazenyl)-2*H*-chromen-2-one (6a). Red microcrystals, mp 201-203°C; ^{1}H-NMR (DMSO-*d*$_6$) δ: 2.25 (s, 3H, CH_3), 2.61 (s, 3H, CH_3), 6.96-8.48 (m, 14H, Ar-H), 10.36 (s, br, 1H, NH) ppm; ^{13}C-NMR (DMSO-*d*$_6$): δ 8.4, 14.7 (CH_3), 116.3, 118.7, 119.9, 120.1, 123.8, 123.9, 125.0, 129.9, 130.2, 132.9, 136.2, 141.9, 142.7, 147.9, 151.1, 152.6, 154.6, 156.5, 160.0, 162.1 (Ar-C and C=N), 163.5 (C=O) ppm; IR (KBr) ν cm^{-1}: 3374 (NH), 1725 (C=O), 1606 (C=N); MS m/z (%): 507.13 (M^+, 51). Anal. calcd for $C_{27}H_{21}N_7O_2S$ (507.57): C, 63.89; H, 4.17; N, 19.32. Found: C, 63.77; H, 4.03; N, 19.20%.

3-(1-(2-(4-Methyl-5-(p-tolyldiazenyl)thiazol-2-yl)hydrazineylidene)ethyl)-6-(phenyldiazenyl)-2*H*-chromen-2-one (6b). Orange microcrystals, mp 193-195 °C; ^{1}H-NMR (DMSO-*d*$_6$) δ: 2.26 (s, 3H, CH_3), 2.46 (s, 3H, CH_3), 2.60 (s, 3H, CH_3), 7.15-8.54 (m, 13H, Ar-H), 10.64 (s, br, 1H, NH) ppm; IR (KBr) ν cm^{-1}: 3371 (NH), 1721 (C=O), 1603 (C=N); MS m/z (%): 521 (M^+, 47). Anal. calcd for $C_{28}H_{23}N_7O_2S$ (521.60): C, 64.48; H, 4.44; N, 18.80. Found: C, 64.27; H, 4.36; N, 18.69%.

3-(1-(2-(5-((4-Methoxyphenyl)diazenyl)-4-methylthiazol-2-yl)hydrazineylidene)ethyl)-6-(phenyldiazenyl)-2*H*-chromen-2-one (6c). Red microcrystals, mp 179-181°C; ^{1}H-NMR (DMSO-d_6) δ: 2.23 (s, 3H, CH_3), 2.42 (s, 3H, CH_3), 3.82 (s, 3H, OCH_3), 6.86-8.19 (m, 13H, Ar-H), 10.38 (s, br, 1H, NH) ppm; ^{13}C-NMR (DMSO-d_6): δ 9.1, 14.7 (CH_3), 56.5 (OCH_3), 114.1, 115.8, 119.8, 121.9, 122.3, 125.9, 128.6, 129.8, 130.1, 132.1, 134.1, 136.4, 138.3, 139.8, 142.7, 146.7, 149.6, 149.7, 150.0, 158.2 (Ar-C and C=N), 164.1 (C=O) ppm; IR (KBr) ν cm^{-1}: 3338 (NH), 1725 (C=O), 1605 (C=N); MS m/z (%): 537 (62). Anal. calcd for $C_{28}H_{23}N_7O_3S$ (537.60): C, 62.56; H, 4.31; N, 18.24. Found: C, 62.47; H, 4.19; N, 18.07%.

3-(1-(2-(5-((4-Chlorophenyl)diazenyl)-4-methylthiazol-2-yl)hydrazineylidene)ethyl)-6-(phenyldiazenyl)-2*H*-chromen-2-one (6d). Red microcrystals, mp 207-209 °C; ^{1}H-NMR (DMSO-d_6) δ: 2.24 (s, 3H, CH_3), 2.42 (s, 3H, CH_3), 6.54-8.81 (m, 13H, Ar-H), 10.73 (s, br, 1H, NH) ppm; IR (KBr) ν cm^{-1}: 3377 (NH), 1728 (C=O), 1609 (C=N); MS m/z (%): 544 (M^++2, 8), 542 (M^+, 30). Anal. calcd for $C_{27}H_{20}ClN_7O_2S$ (542.01): C, 59.83; H, 3.72; N, 18.09. Found: C, 59.69; H, 3.58; N, 18.01%.

3-(1-(2-(5-((2,4-Dichlorophenyl)diazenyl)-4-methylthiazol-2-yl)hydrazineylidene)ethyl)-6-(phenyldiazenyl)-2*H*-chromen-2-one (6e). Dark red microcrystals, mp 216-218 °C; ^{1}H-NMR (DMSO-d_6) δ: 2.42 (s, 3H, CH_3), 6.86-8.52 (m, 12H, Ar-H), 10.37 (s, br, 1H, NH) ppm; IR (KBr) ν cm^{-1}: 3380 (NH), 1729 (C=O), 1613 (C=N); MS m/z (%): 576 (M^+, 49). Anal. calcd for $C_{27}H_{19}Cl_2N_7O_2S$ (576.46): C, 56.26; H, 3.32; N, 17.01. Found: C, 56.13; H, 3.25; N, 16.86%.

2-(2-(1-(2-oxo-6-(Phenyldiazenyl)-2*H*-chromen-3-yl)ethylidene)hydrazineyl)-5-(2-(p-tolyl)hydrazineylidene)thiazol-4(5H)-one (10a). Yellow microcrystals, mp 178-170°C; ^{1}H-NMR (DMSO-d_6) δ: 2.26 (s, 3H, CH_3), 2.27 (s, 3H, CH_3), 7.02-7.92 (m, 13H, Ar-H), 10.54 (s, br, 1H, NH), 10.79 (s, br, 1H, NH) ppm; ^{13}C-NMR (DMSO-d_6): δ 8.9, 14.2 (CH_3), 117.0, 118.8, 120.4, 122.5, 122.8, 123.0, 124.2, 127.0, 129.3, 129.5, 129.7, 129.8, 129.9, 130.0, 138.5, 142.1, 145.2, 152.3, 156.7 (Ar-C and C=N), 163.0, 171.3 (C=O) ppm; IR (KBr) ν cm^{-1}: 3413, 3289 (2NH), 1724, 1692 (2C=O), 1604 (C=N); MS m/z (%): 523 (M^+, 100). Anal. calcd for $C_{27}H_{21}N_7O_3S$ (523.57): C, 61.94; H, 4.04; N, 18.73. Found: C, 61.94; H, 4.04; N, 18.73%.

5-(2-(4-Chlorophenyl)hydrazineylidene)-2-(2-(1-(2-oxo-6-((phenyldiazenyl)-2*H*-chromen-3-yl)ethylidene)hydrazineyl)thiazol-4(5*H*)-one (10b). Yellow microcrystals, mp 190-192°C; ^{1}H-NMR (DMSO-d_6) δ: 2.20 (s, 3H, CH_3), 7.03-7.93 (m, 13H, Ar-H), 10.38 (s, br, 1H, NH), 10.70 (s, br, 1H, NH) ppm; IR (KBr) ν cm^{-1}: 3439, 3277 (2NH), 1725, 1697 (2C=O), 1606 (C=N); MS m/z (%): 545 (M^++2, 25), 543 (M^+, 79). Anal. calcd for $C_{26}H_{18}ClN_7O_3S$ (543.99): C, 57.41; H, 3.34; N, 18.02. Found: C, 57.36; H, 3.18; N, 17.85%.

5-(2-(4-Nitrophenyl)hydrazineylidene)-2-(2-(1-(2-oxo-6-(phenyldiazenyl)-2*H*-chromen-3-yl)ethylidene)hydrazineyl)thiazol-4(5*H*)-one (10c). Brown microcrystals, mp 178-170°C; ^{1}H-NMR (DMSO-d_6) δ: 2.32 (s, 3H, CH_3), 7.50-8.34 (m, 13H, Ar-H), 10.38 (s, br, 1H, NH), 11.09 (s, br, 1H, NH) ppm; IR (KBr) ν cm^{-1}: 3435, 3297 (2NH), 1727, 1704 (2C=O), 1616 (C=N); MS m/z (%): 554 (M^+, 64). Anal. calcd for $C_{26}H_{18}N_8O_5S$ (554.54): C, 56.31; H, 3.27; N, 20.21. Found: C, 56.19; H, 3.06; N, 20.14%.

6-(Phenyldiazenyl)-3-(1-(2-(4-phenylthiazol-2-yl)hydrazineylidene)ethyl)-2*H*-chromen-2-one (12a). Yellow microcrystals, mp 217-219 °C; ^{1}H-NMR (DMSO-*d*$_6$) *δ*: 2.31 (s, 3H, CH_3), 6.94-8.42 (m, 14H, Ar-H), 11.32 (s, br, 1H, NH) ppm; ^{13}C-NMR (DMSO-*d*$_6$): *δ* 10.1(CH_3), 106.1, 116.5, 116.6, 117.9, 119.8, 120.0, 124.6, 125.3, 125.8, 129.5, 129.6, 131.6, 133.1, 136.5, 139.9, 143.4, 144.0, 150.8, 153.8, 159.6 (Ar-C and C=N), 164.5 (C=O) ppm; IR (KBr) *ν* cm^{-1}: 3327 (NH), 1722 (C=O), 1609 (C=N); MS m/z (%): 465 (M$^+$, 91). Anal. calcd for $C_{26}H_{19}N_5O_2S$ (465.53): C, 67.08; H, 4.11; N, 15.04. Found: C, 67.15; H, 4.03; N, 14.91%.

3-(1-(2-(4-(4-Methoxyphenyl)thiazol-2-yl)hydrazineylidene)ethyl)-6-(phenyldiazenyl)-2*H*-chromen-2-one (12b). Yellow microcrystals, mp 205-207 °C; ^{1}H-NMR (DMSO-*d*$_6$) *δ*: 2.30 (s, 3H, CH_3), 3.82 (s, 3H, OCH_3), 7.25-8.38 (m, 13H, Ar-H), 11.53 (s, br, 1H, NH) ppm; IR (KBr) *ν* cm^{-1}: 3366 (NH), 1723 (C=O), 1602(C=N); MS m/z (%): 495 (M$^+$, 58). Anal. calcd for $C_{27}H_{21}N_5O_3S$ (495.56): C, 65.44; H, 4.27; N, 14.13. Found: C, 65.30; H, 4.13; N, 14.04%.

3-(1-(2-(4-(4-Nitrophenyl)thiazol-2-yl)hydrazineylidene)ethyl)-6-(phenyldiazenyl)-2*H*-chromen-2-one (12c). Brown microcrystals, mp 231-233 °C; ^{1}H-NMR (DMSO-*d*$_6$) *δ*: 2.31 (s, 3H, CH_3), 7.62-8.44 (m, 13H, Ar-H), 11.59 (s, br, 1H, NH) ppm; IR (KBr) *ν* cm^{-1}: 3373 (NH), 1724 (C=O), 1606 (C=N); MS m/z (%): 510 (M$^+$, 37). Anal. calcd for $C_{26}H_{18}N_6O_4S$ (510.53): C, 61.17; H, 3.55; N, 16.46. Found: C, 61.03; H, 3.48; N, 16.35%.

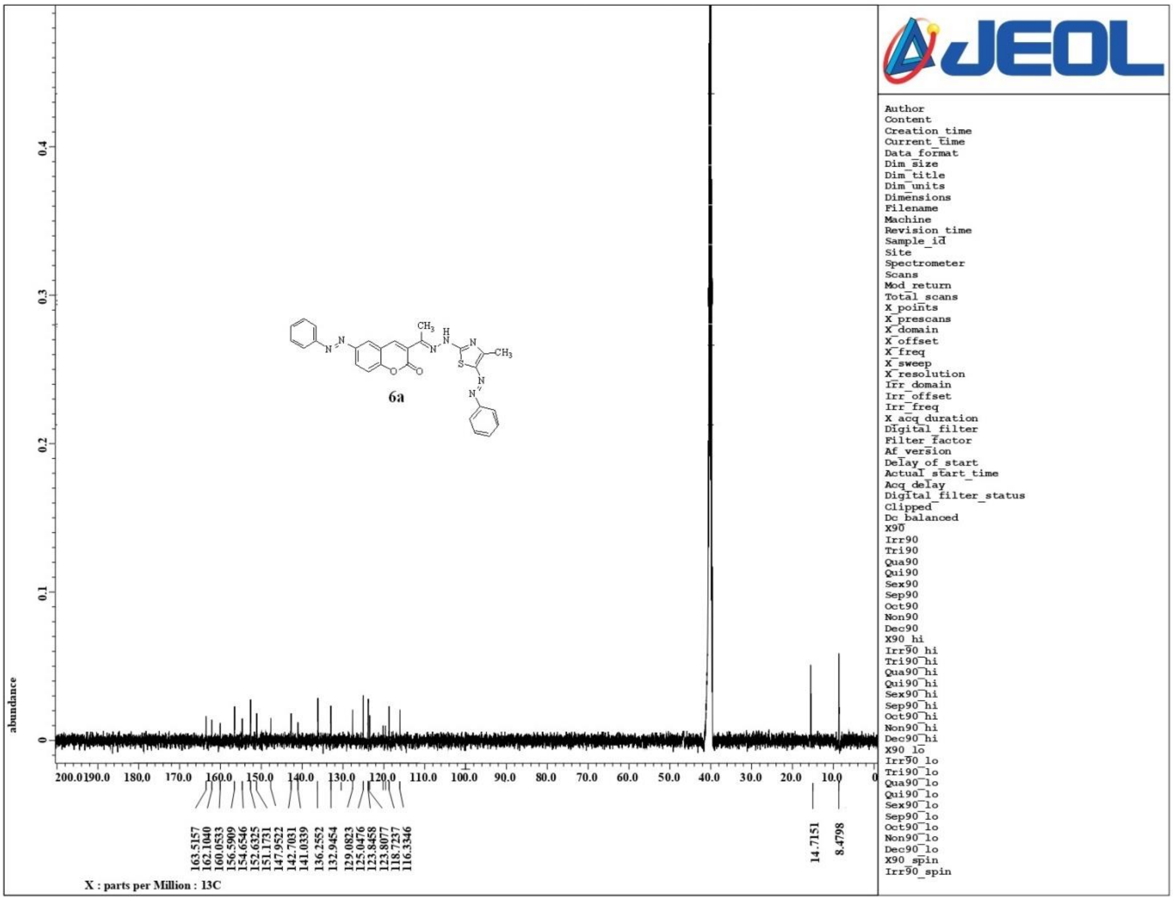

^{13}C-NMR spectra of compound **6a**

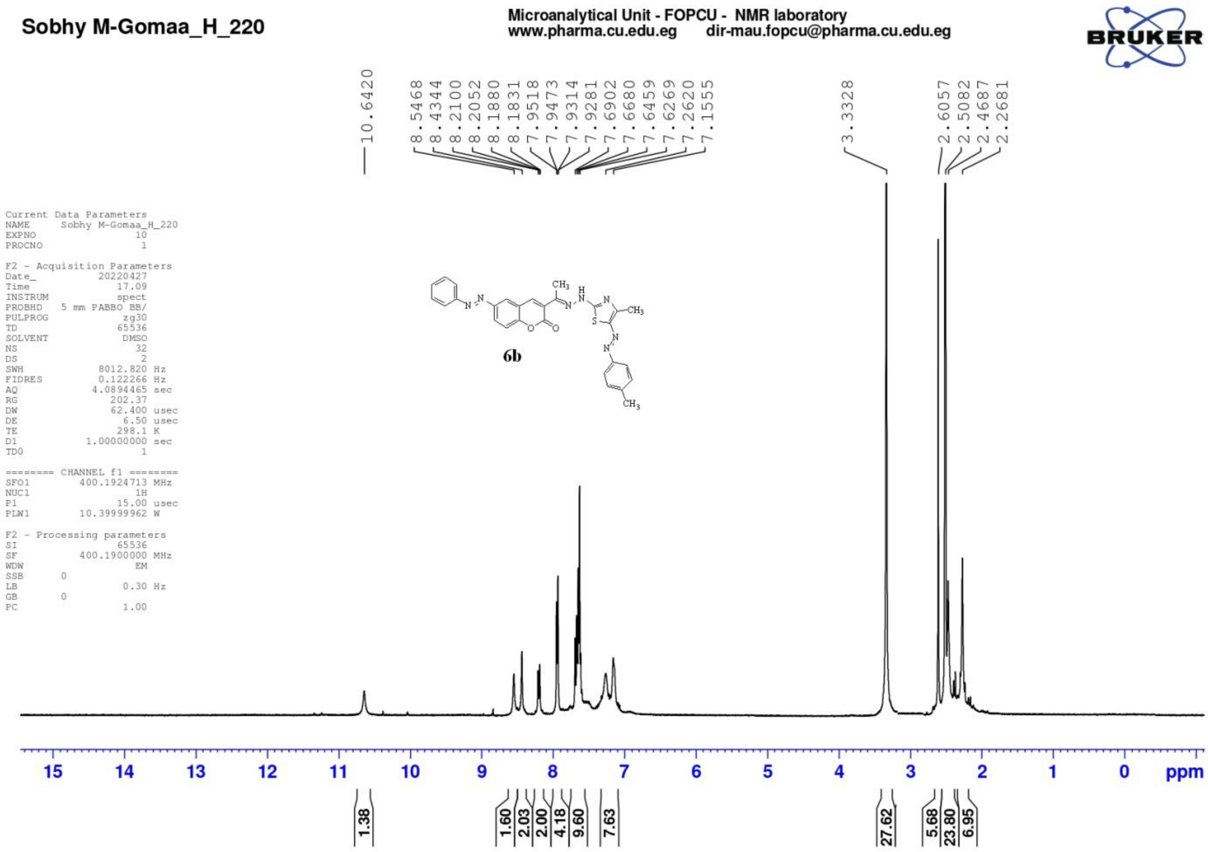

^{1}H-NMR spectra of compound **6b**

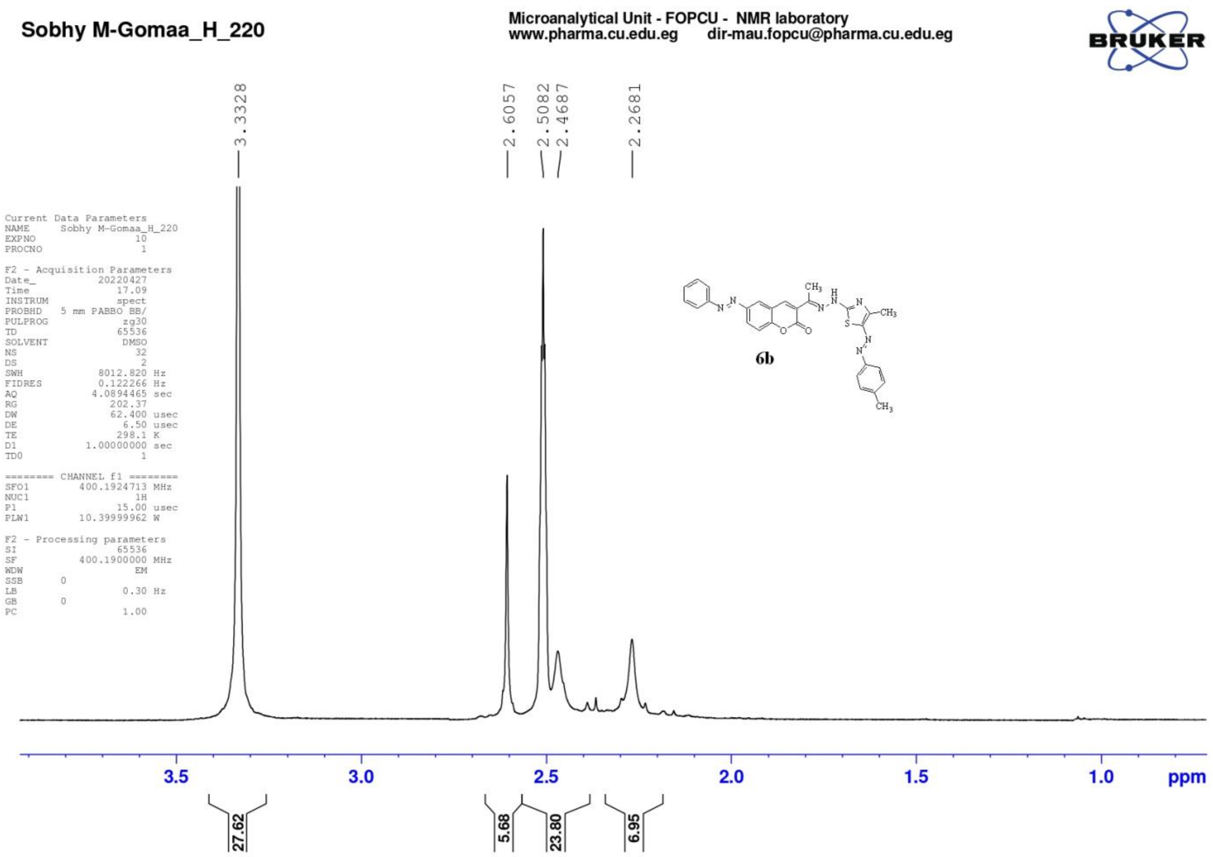

^{1}H-NMR spectra of compound **6b-1**

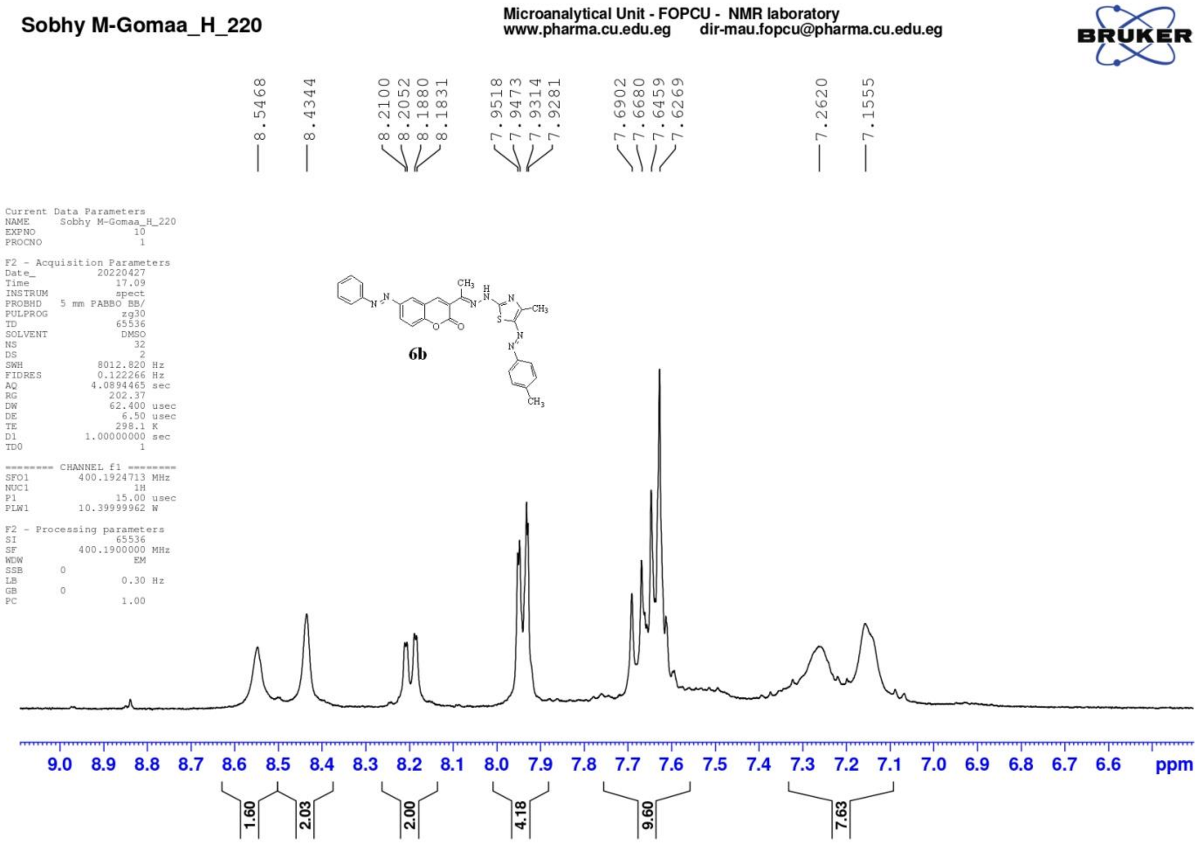

^{1}H-NMR spectra of compound **6b-2**

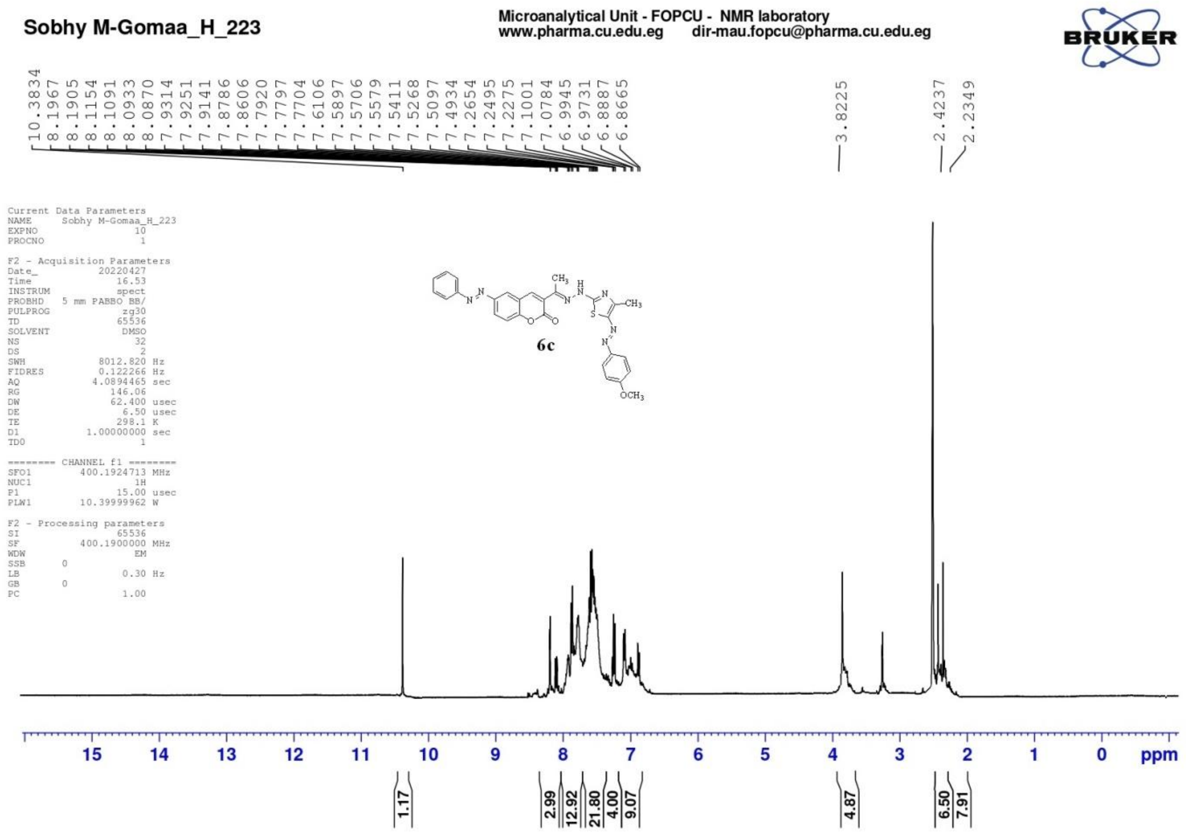

^{1}H-NMR spectra of compound **6c**

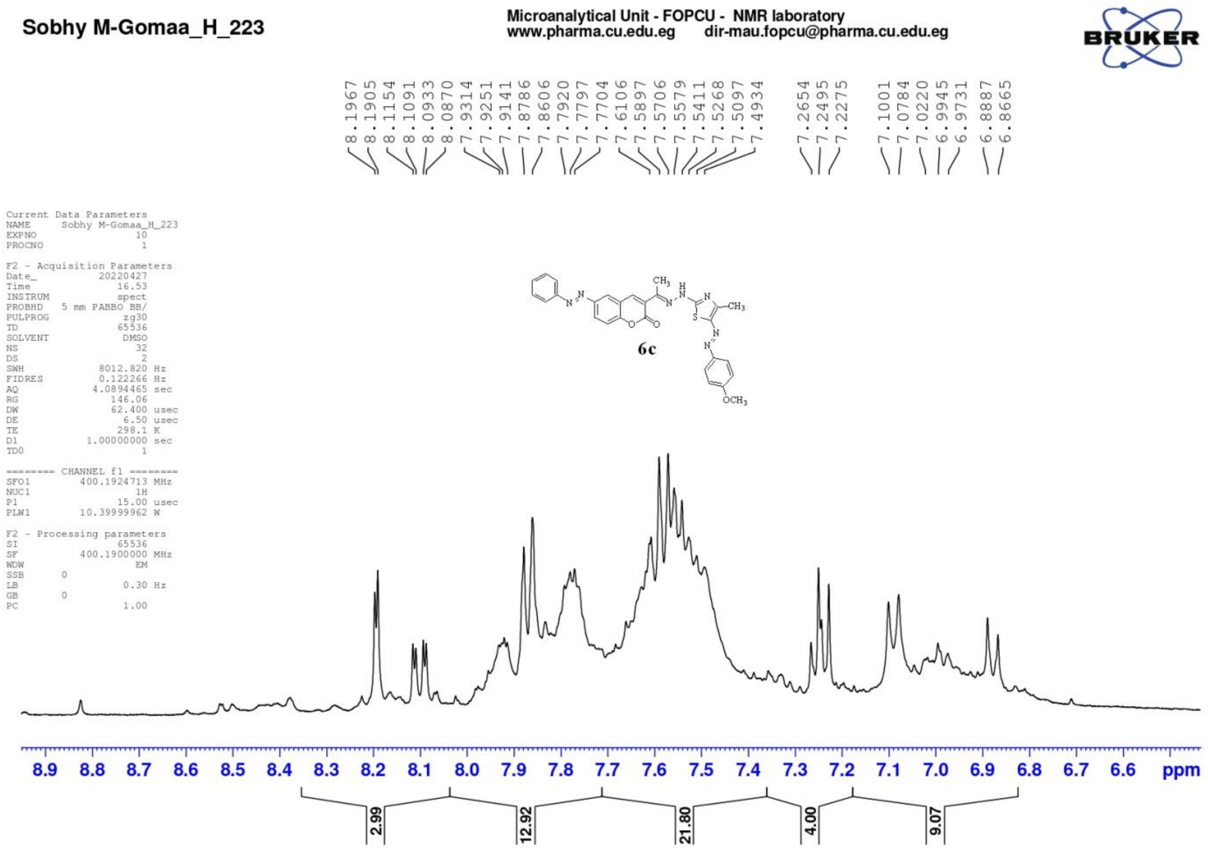

^{1}H-NMR spectra of compound **6c-2**

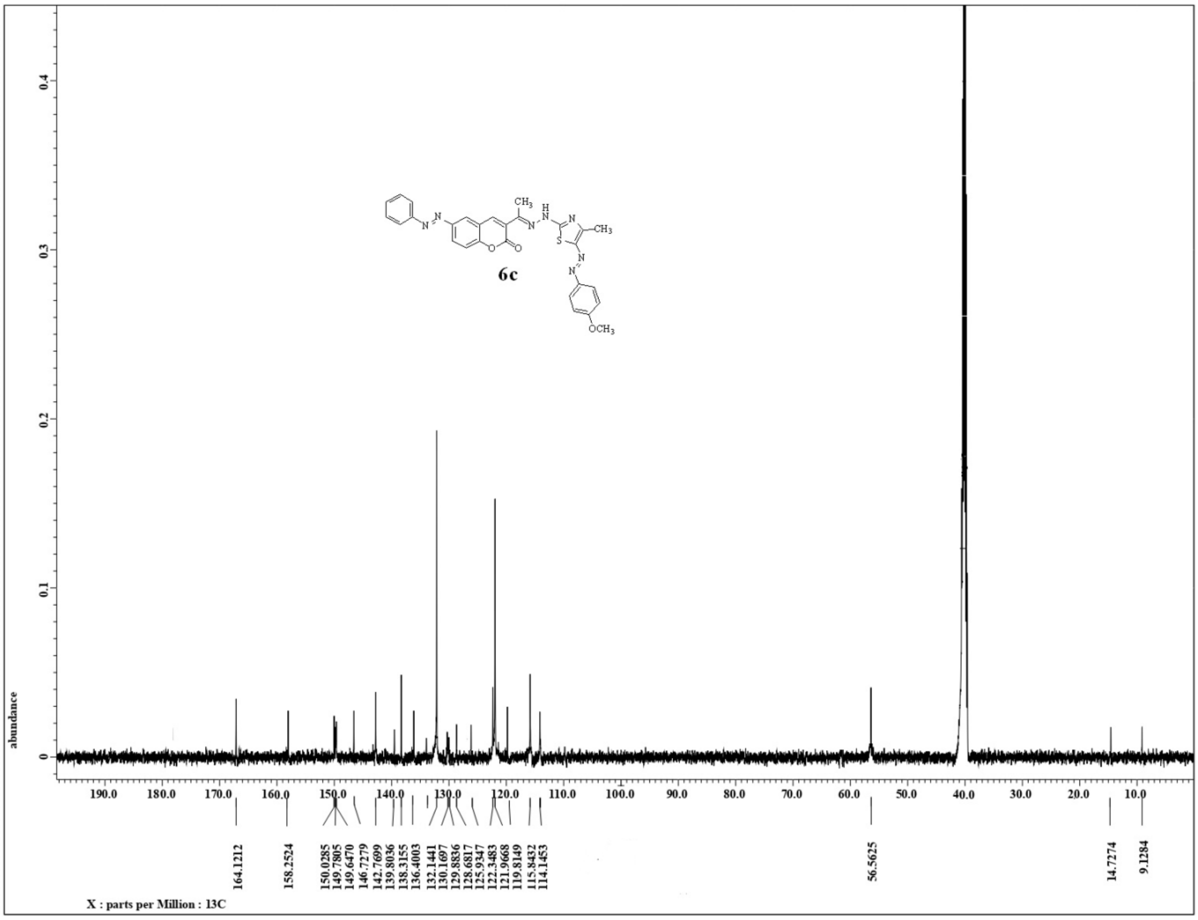

^{13}C-NMR spectra of compound **6c**

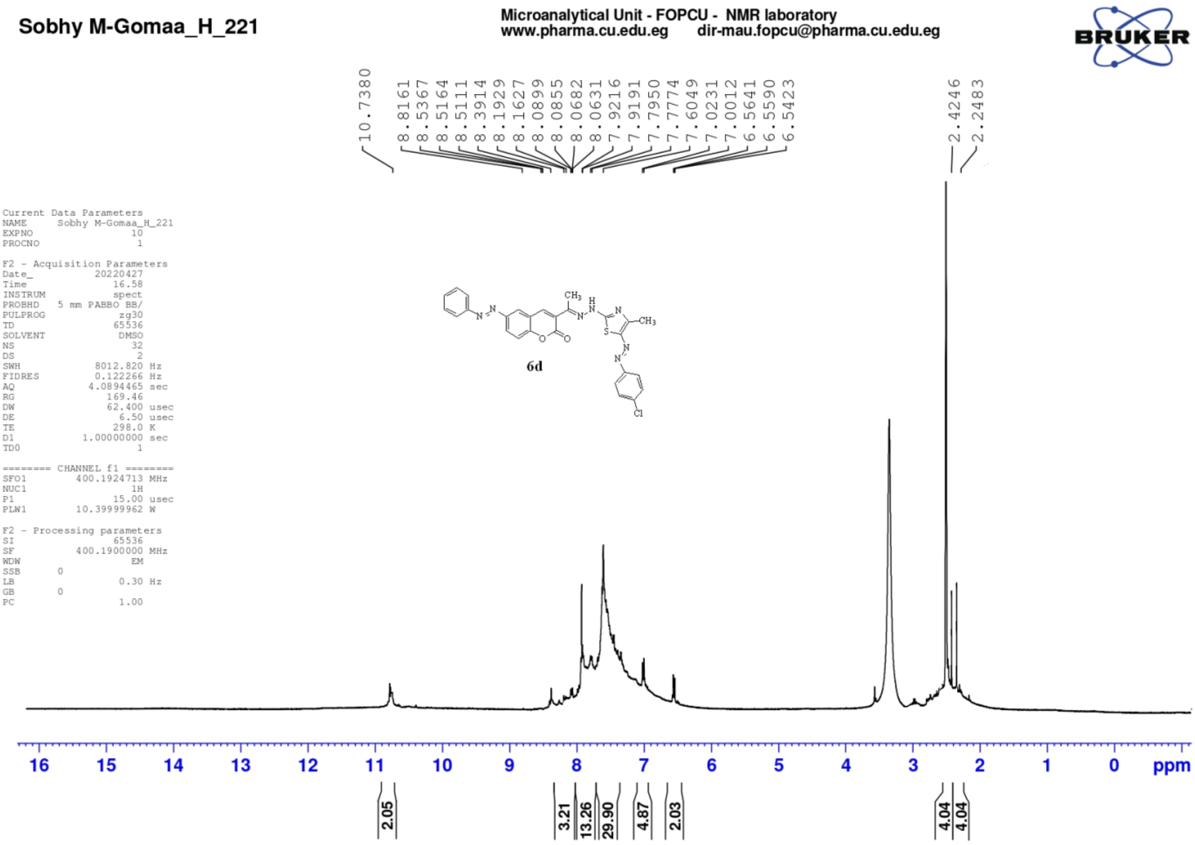

^{1}H-NMR spectra of compound **6d**

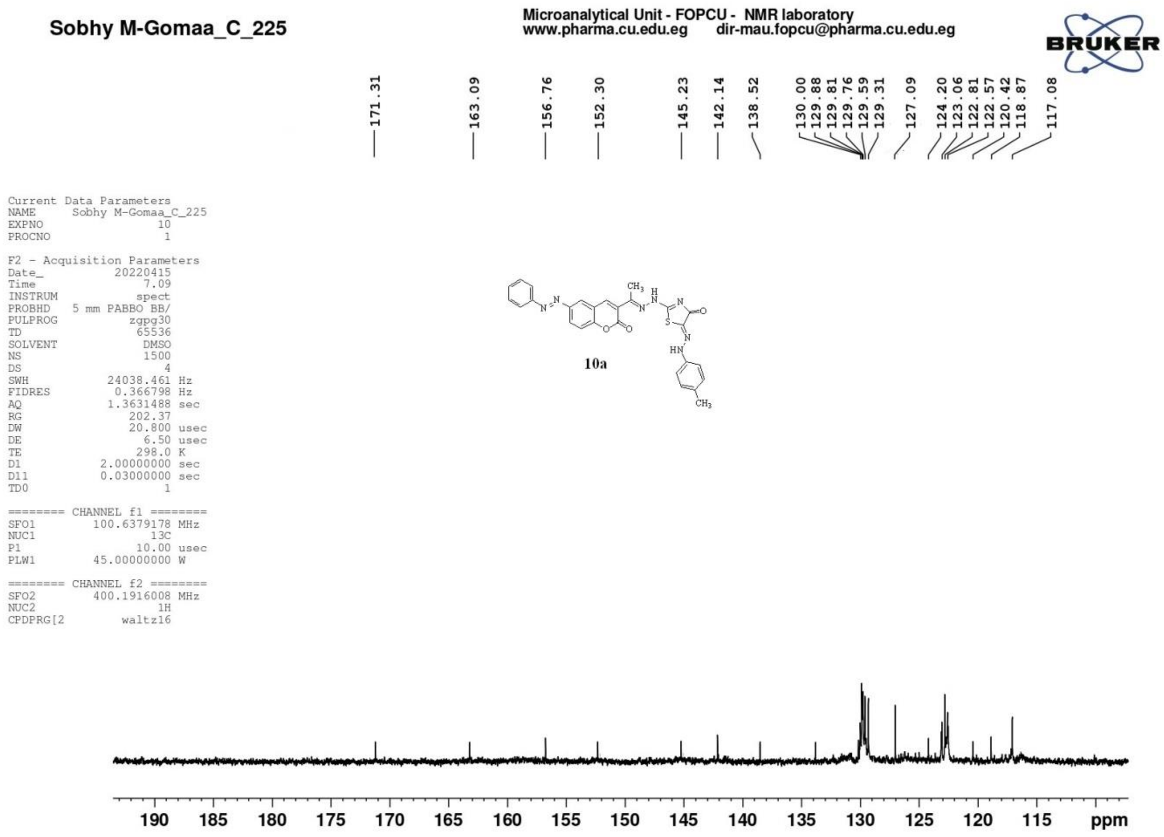

^{13}C-NMR spectra of compound **10a**

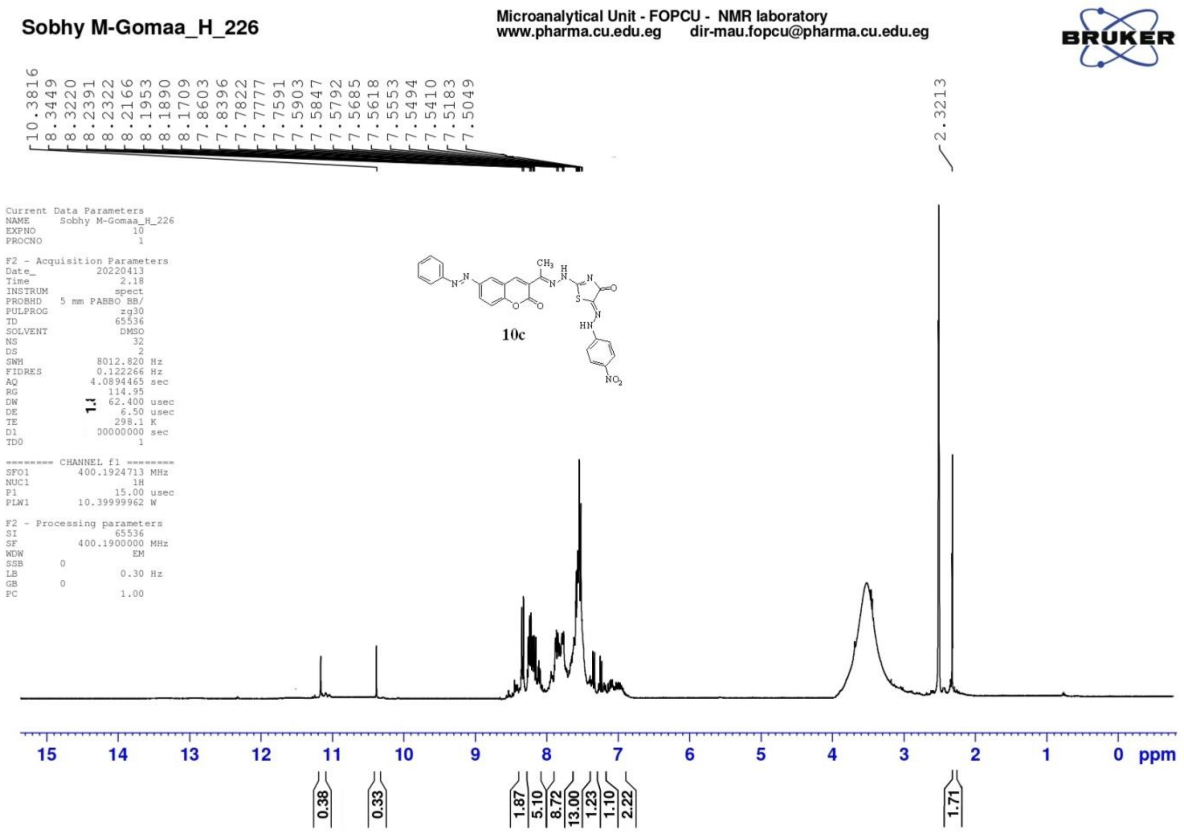

^{1}H-NMR spectra of compound **10c**

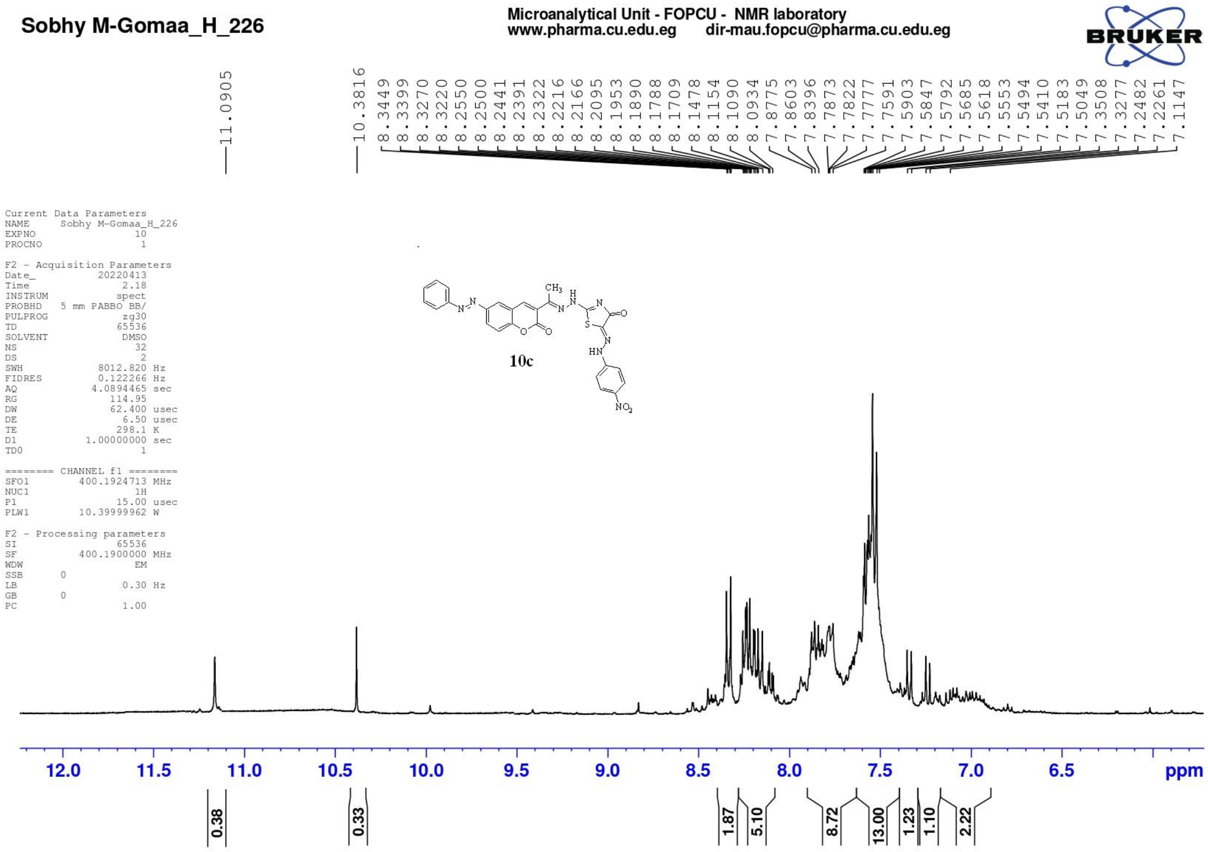

^{1}H-NMR spectra of compound **10c-2**

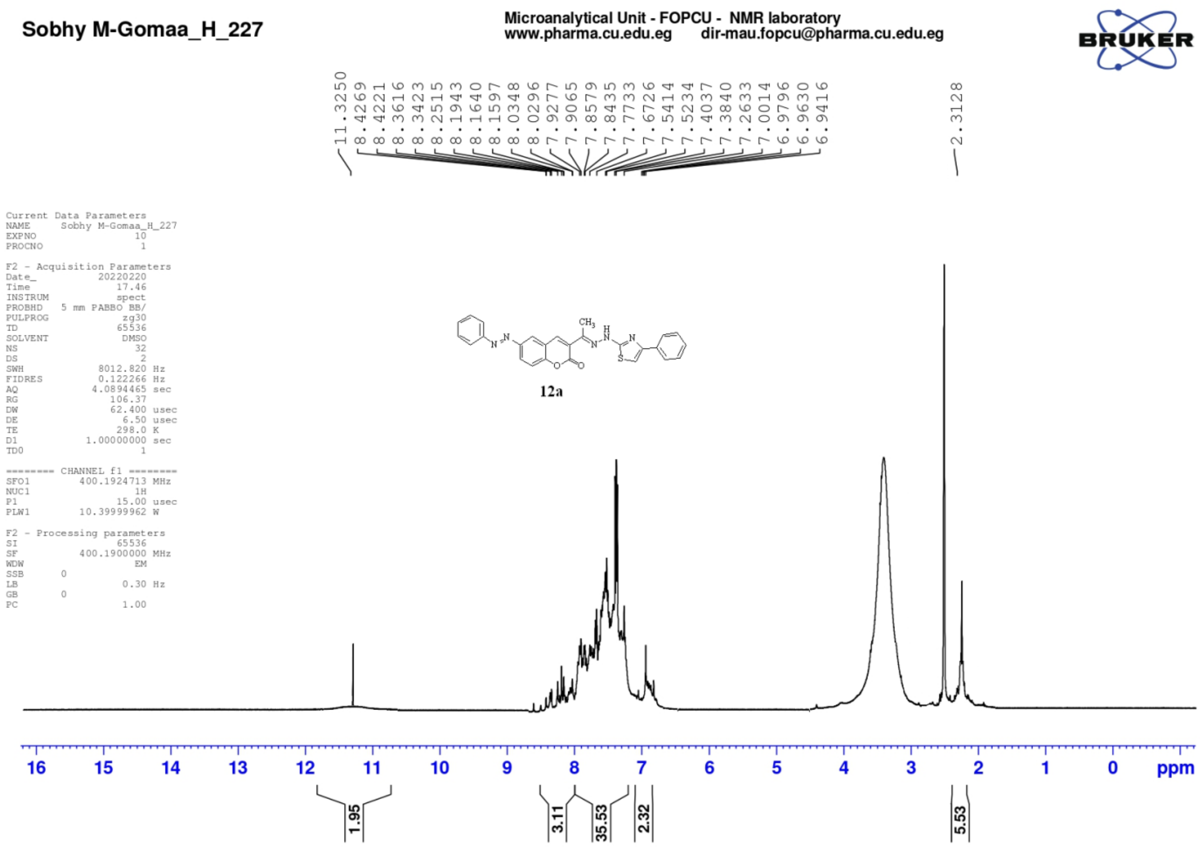

^{1}H-NMR spectra of compound **12a**

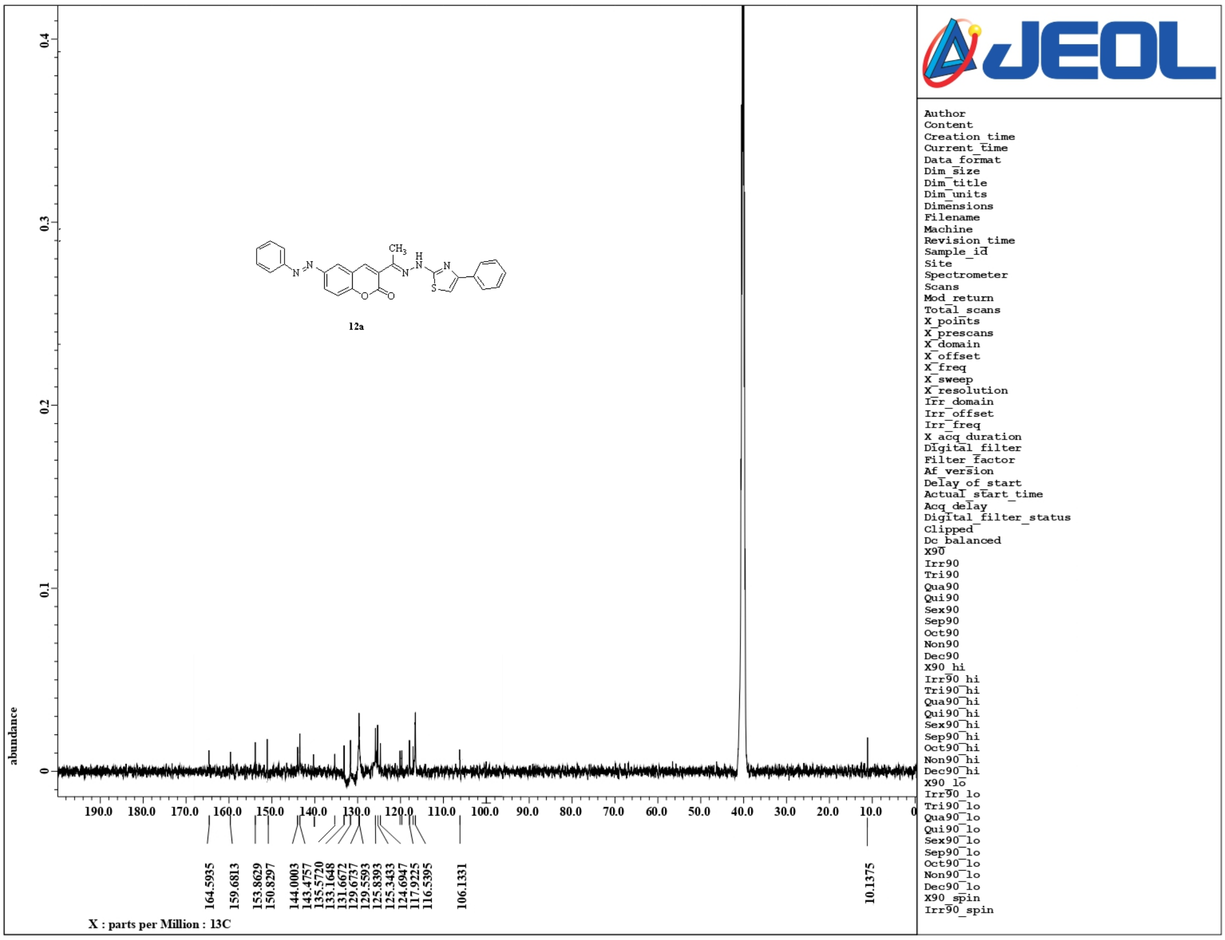

^{13}C-NMR spectra of compound **12a**

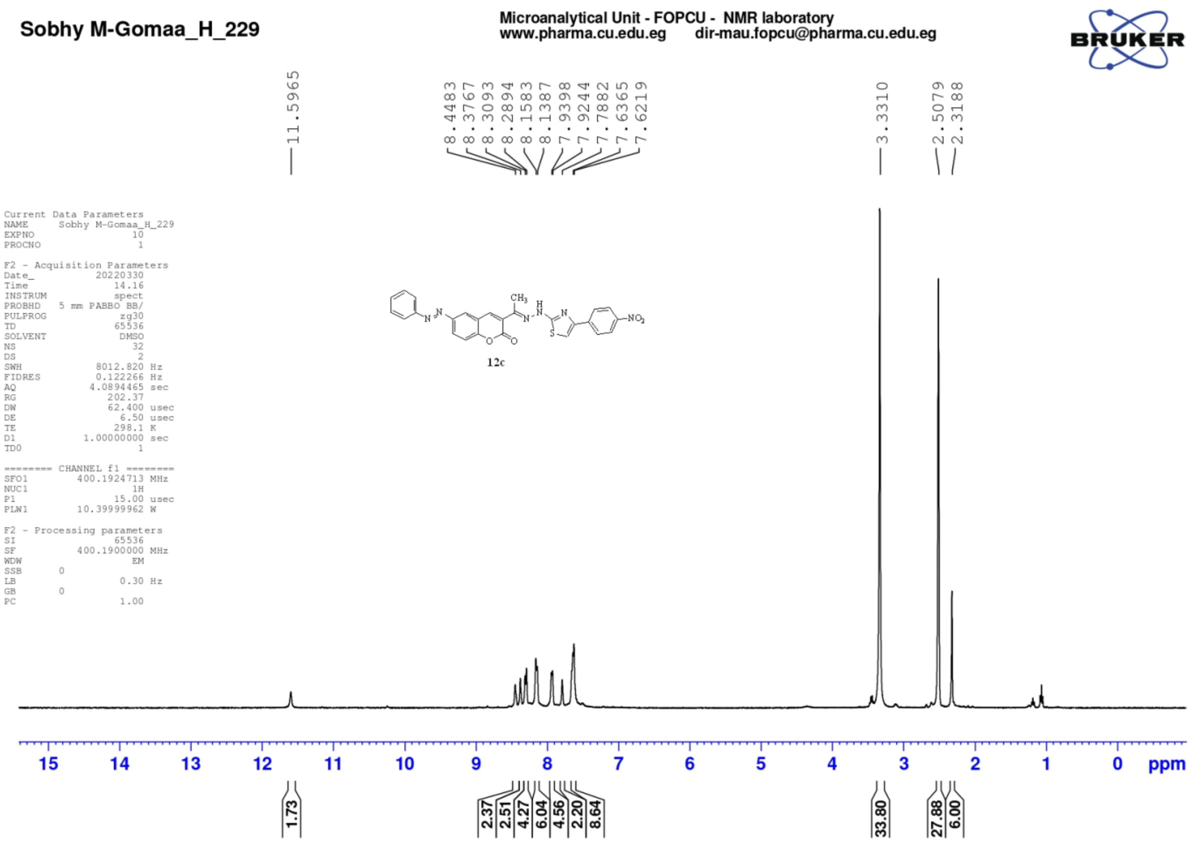

^{1}H-NMR spectra of compound **12c**

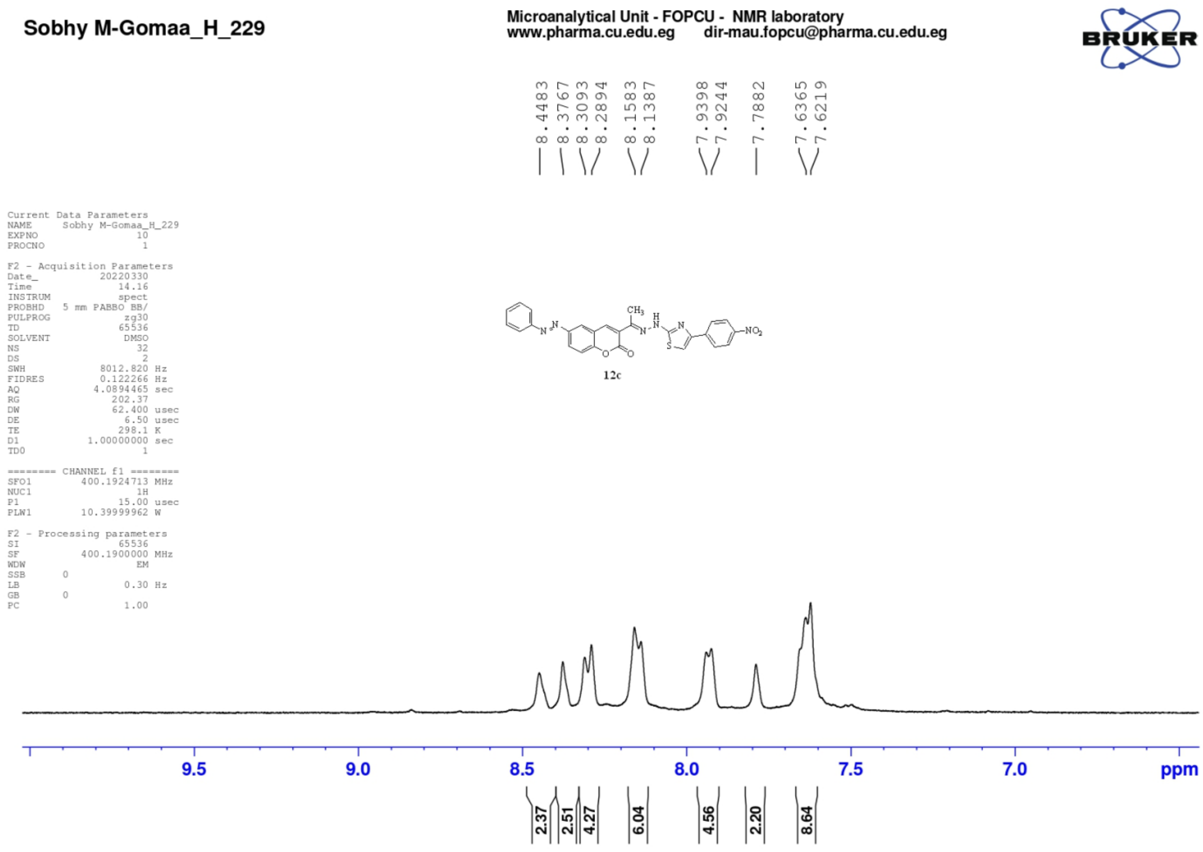

^{1}H-NMR spectra of compound **12c-1**

Cytotoxicity assay:

The tested human breast cancer (MCF-7) cell lines and normal cell line LLC-Mk2 were obtained from the American Type Culture Collection (ATCC, Rockville, MD). The cells were grown on RPMI-1640 medium supplemented with 10% inactivated fetal calf serum and 50μg/mL gentamycin (Lonza, Belgium). The cells were maintained at 37ºC in a humidified atmosphere with 5% CO_2 and were subcultured two to three times a week during the period of experiment .

For antitumor assays, the tumor cell lines were suspended in medium at cell density of $5x10^4$ cells/well in Corning® 96-well tissue culture plates and then incubated for 24 hours. The tested compounds were then added into 96-well plates (six replicates) to achieve eight concentrations for each compound. Six vehicle controls with media or 0.5 % DMSO were run for each 96 well plate as a control. After incubating for 24 h, the numbers of viable cells were determined by the MTT assay [1]. Briefly, the media was removed from the 96-well plate and replaced with 100 μl of fresh culture RPMI 1640 medium without phenol red then 10

μL of the 12 mM MTT stock solution {5 mg of 3-(4,5-dimethylthiazol-2-yl)-2,5-diphenyltetrazolium bromide purchased from Sigma-Aldrich (St. Louis, MO) in 1 mL of Phosphate buffered saline} to each well including the untreated controls. The 96 well plates were then incubated at 37°C and 5% CO_2 for 4 hours. An 85 μl aliquot of the media was removed from the wells, and 50 μL of DMSO was added to each well and mixed thoroughly with the pipette and incubated at 37 °C for 10 min. Then, the optical density was measured at 590 nm with the microplate reader (SunRise, TECAN, Inc, USA) to determine the number of viable cells and the percentage of viability was calculated as [(ODt/ODc)]x100% where ODt is the mean optical density of wells treated with the tested sample and ODc is the mean optical density of untreated cells. The relation between surviving cells and drug concentration is plotted to get the survival curve of each tumor cell line after treatment with the specified compound. The 50% inhibitory concentration (IC_{50}), the concentration required to cause toxic effects in 50% of intact cells, was estimated from graphic plots of the dose response curve for each conc. using GraphPad Prism software (San Diego, CA. USA)[2].

1. Mosmann, T. Rapid colorimetric assay for cellular growth and survival: application to proliferation and cytotoxicity assays. *J. Immunol. Methods* **1983**, *65*, 55-63.
2. Gomha, S.M.; Riyadh, S.M.; Mahmoud, E.A.; Elaasser, M.M. Synthesis and anticancer activities of thiazoles, 1,3-thiazines, and thiazolidine using chitosan-grafted-poly(vinylpyridine) as basic catalyst. *Heterocycles* **2015**, *91*, 1227-1243.
